# Book Reviews

**Published:** 1892-02

**Authors:** 


### Book Reviews.

AN ABSTRACT OF THE SYMPTOMS, with the Latest Dietetic and Medical Treatment of Various Diseased Conditions. New York. Reed and Carnrick.

This convenient and useful little book contains a great deal of valuable information, particularly with reference to the dietary of the sick. Of course the preparations of this manufacturing house are given considerable prominence. In Part I. concise outlines of the leading symptoms of the common diseases are presented, also the dietetic and medicinal treatment that is most effectual in relieving or curing these abnormal conditions. Part II. is an excellent contribution to the subject of the physiology of digestion and assimilation, and deserves a careful reading.

---

INTERNATIONAL CLINICS: A Quarterly of Clinical Lectures October, 1891. J. B. Lippincott Company, Philadelphia.

This is the third volume of a valuable publication which has been favorably noticed before in this journal. The present fully sustains the excellence of those which have preceded it, and, like the others, furnishes clinical lectures in nearly every department of medical and surgical practice.

---

MEDICAL REVIEW VISITING LIST.—St. Louis, Mo., J. H. Chambers & Co.; price 75 cents.

This book contains a perpetual visiting list with a pocket reference book. The pages for weekly call and memoranda are ample and the tables for ready reference are both valuable and convenient.

---

The St. Louis *Medical Review* comes out for 1892 in a new attire, under the editorial control of Dr. A. H. Ohmann-Dumesnil. Many have been the changes in the staff of the *Review* during the year just past. The present management seems to have struck out on a new line, and we wish for it a long and abiding success.

ST. LOUIS is nothing if not prolific in medical journals. On January first the *Medical Fortnightly* appeared, under the editorship of Dr. Bransford Lewis. The first issue of the *Fortnightly* is very creditable. The matter is good and the printers' work is very well executed. The *Fortnightly* is seized in the beginning with the high and noble ambition of trying to pacify the discordant elements of the St. Louis profession. This is an undertaking as praiseworthy as it is immense. The *Fortnightly* will proceed to be a "magnet which will draw together into happy and felicitous union this array of otherwise-divided medical teachers."

The names of several of the St. Louis brethren are harmoniously commingled among the collaborators of the *Fortnightly*, and we presume they are now working together with unity of spirit and purpose. It is a blessed thing for brethren to dwell together in unity. Let us all pray!

---

ONE of the most interesting and excellent periodicals that is gotten out by the enterprise and competition of American publishers is Lippincott's Magazine. For a quarter of a century it has enjoyed the patronage and esteem of a large number of readers, and to-day it is easily one of the best of our literary monthlies. The characteristic feature of this magazine is the appearance, in every issue, of a complete novel, by some prominent living author. In recent numbers these have been furnished by Julian Hawthorne, Edgar Fawcett, Lloyd Brice, Mrs. Alexander, Rudyard Kipling, and others of equal note. This year the magazine will contain novels by Marion Harland, Capt. Chas. King, Frances Courtenay Baylor, Amelia Barr and others.

In addition to this, a most valuable feature will be a series of articles dealing with reminiscences of men famous in our political history, as, Abraham Lincoln, Jno. C. Calhoun, Andrew Johnson, etc. "Lippincott's" deserves the cordial patronage and appreciation of all lovers of good literature.

